# Severity score for hereditary hemorrhagic telangiectasia

**DOI:** 10.1186/s13023-014-0188-3

**Published:** 2014-12-29

**Authors:** Giuseppe A Latino, Helen Kim, Jeffrey Nelson, Ludmila Pawlikowska, William Young, Marie E Faughnan, the Brain Vascular Malformation Consortium HHT Investigator Group

**Affiliations:** Department of Pediatrics, The Hospital for Sick Children, University of Toronto, 555 University Avenue, Toronto, ON M5G 1X8 Canada; Division of Respirology, Department of Medicine, St. Michael’s Hospital, University of Toronto, Toronto, Canada; Center for Cerebrovascular Research, Department of Anesthesia and Perioperative Care, University of California, San Francisco, USA; Institute for Human Genetics, University of California, San Francisco, USA; Li Ka Shing Knowledge Institute of St. Michaels Hospital, Toronto, Canada

**Keywords:** Hereditary Hemorrhagic Telangiectasia (HHT), Osler-Weber-Rendu, Arteriovenous Malformation (AVM), Bleeding, Disease severity, Severity score

## Abstract

**Background:**

A disease severity score in hereditary hemorrhagic telangiectasia (HHT) would be a useful tool for assessing burden of disease and for designing clinical trials. Here, we propose the first known HHT severity score, the HHT-score.

**Methods:**

Demographics and disease characteristics were collected for the first 525 HHT patients recruited to the HHT Project of the Brain Vascular Malformation Consortium (BVMC). HHT-score was calculated based on presence of: organ arteriovenous malformations (maximum 3 points); chronic bleeding (maximum 2 points); and severe organ involvement (maximum 2 points). Points were summed and patients categorized as having mild (0–2), moderate (3–4) or severe (5–7) disease. The occurrence of “any adverse outcome” was evaluated for association with HHT-score categories.

**Results:**

The frequency of “any adverse outcome” was significantly different across the three groups (49.6% in mild, 65.8% in moderate and 89.5% in severe, p < 0.001). Adjusting for age and gender, the risk of “any adverse outcome” was higher in the moderate (OR = 1.84, 95% CI: 1.15-2.95, p = 0.011) and severe groups (OR = 9.16, 95% CI: 1.99-42.09, p = 0.004) compared to the mild.

**Conclusions:**

We have taken the first steps toward creating a global measure of disease severity in HHT. While the initial results are promising, further validation of the HHT-score is still required.

## Background

Hereditary hemorrhagic telangiectasia (HHT) is an autosomal dominant condition of vascular dysplasia characterized by mucocutaneous telangiectases and organ arteriovenous malformations (AVMs) mainly affecting the lungs, liver and brain [1,2]. To date, mutations in three different genes have been identified, all coding for proteins of the TGF-β/BMP-9 signalling pathway. The two most commonly mutated genes are endoglin (*ENG* or HHT1) and activin A receptor type II-like 1 (*ALK-1* or HHT2) [1], with *SMAD4* mutations reported in a small subset of patients, most of whom also have juvenile polyposis [1,3]. HHT is thought to affect at least 1 in 10000 individuals with no known ethnic predisposition [1,2,4-8].

Telangiectases and AVMs are prone to bleeding, leading to chronic epistaxis and/or gastrointestinal (GI) bleeding in most HHT patients, and a risk of life-threatening pulmonary or cerebral hemorrhage in approximately 50% [1,9,10]. Patients may also develop potentially fatal complications from arteriovenous shunting, including stroke, brain abscess and high-output cardiac failure [1,9,10].

While the penetrance of HHT is very high, there is wide variability in disease expression and severity [1]. To date, there is no global measure of disease severity in HHT, which affects multiple organ systems. Although an epistaxis severity score (ESS) is available for this symptom of HHT [11], there is currently no overall HHT severity score, and yet this would be helpful for assessing burden of disease, for stratifying patients and for research study design. In fact, the development of disease severity measures has been identified as a research priority by the HHT Foundation International [12]. Here, we present the first HHT severity score (HHT-score), incorporating organ involvement and chronic bleeding, and evaluated whether HHT-scores are associated with adverse outcomes.

## Methods

### The Brain Vascular Malformation Consortium (BMVC) HHT project

The Brain Vascular Malformation Consortium (BVMC) is an integrated group of academic medical centers, patient support organizations, and clinical research resources focused on clinical research on brain vascular malformations and improving care for patients with HHT, Sturge-Weber syndrome and Familial Cavernous Malformations. Each of the three study groups has established scalable relational databases for observational and longitudinal studies.

The HHT project of the BVMC represents the first large-scale multicenter global collaboration in HHT research, including fourteen HHT centres of excellence in Canada, the US and Europe. Between June 2010 and January 2013, comprehensive data was collected for 525 recruited HHT patients, 129 of whom have BAVMs. The population characteristics of the cohort are similar to those reported in the literature for unselected HHT populations [13]. While the database was primarily created for the study of BAVMs and ICH, it was intended to also serve as a platform to foster HHT research in other non-BAVM related projects.

### Study population

We performed a retrospective cross-sectional review of the first 525 patients with HHT recruited to the HHT Project of the BVMC. Recruited patients had been screened for organ involvement and other clinical features according to routine clinical practice in HHT Centers of Excellence.

The routine initial assessment of HHT patients seen at the 14 participating HHT Centers included: a comprehensive history and physical for symptoms and complications of HHT, screening for PAVMs (contrast echocardiography and confirmatory computed tomography (CT) for all positive echocardiography results), screening for BAVMs (cerebral magnetic resonance imaging with use of contrast variable among centers), clinical screening for recurrent spontaneous epistaxis (>1 episode per month for >1 year), HHT-related GI-bleeding (anemia, iron deficiency, known GI telangiectases on endoscopy, melena, rectal bleeding), clinical screening for liver VMs (clinical symptoms including chronic right upper quadrant pain, complications such as portal hypertension, high-output heart failure, liver bruit on examination, abnormal liver function tests) as well as routine blood work (complete blood count, ferritin). When initial screening was positive for PAVMs or BAVMs, patients underwent diagnostic imaging and preventative treatment, where appropriate, according to International HHT Guidelines [1]. If initial clinical assessment was suggestive of HHT-related GI bleeding, then diagnostic endoscopy was recommended, and endoscopic, medical and supportive therapies were undertaken on a case-by-case basis. If initial clinical assessment was suggestive of symptomatic liver VMs, then diagnostic imaging was recommended and supportive therapy was also undertaken on a case-by case basis.

Data collected included age, sex, HHT mutation type, clinical presentation and symptoms, presence of AVMs on imaging, and outcomes. Those with incomplete data were excluded, as well as pediatric patients (≤18 years old) because of the small number of patients recruited to date. All patients provided written, informed consent and the study protocol was approved by individual research ethics board at each center.

### HHT-score definition

**Figure 1:**
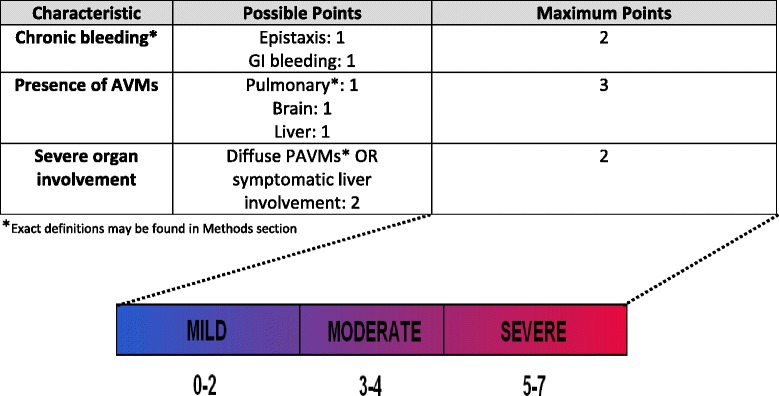
**Definition of the proposed HHT-score.**

An HHT-score was developed based on organ involvement and the presence of chronic bleeding. The score elements were proposed *a priori* from clinical expertise (MEF, GAL) and were assigned a numerical value based on their assumed impact on disease severity (Figure 1). Chronic bleeding was assigned a score of 1 point each for nasal and GI bleeding. A score of 1 point was received for each organ affected by AVMs, up to a maximum of 3 points. The presence of PAVMs was given 1 point only if AVMs were confirmed on CT thorax. Lastly, given the greater perceived impact of certain severe phenotypes of organ involvement on outcomes in HHT, 2 points were given to patients with diffuse PAVMs and/or symptomatic liver involvement. Diffuse PAVMs were defined as AVMs involving every subsegmental artery of at least one lobe.

Total points were tallied for each patient with a possible minimum score of 0 to a maximum of 7. Based on the calculated values, patients were arbitrarily categorized as having mild disease for a score of 0 to 2, moderate disease for a score of 3 to 4, and severe disease for a score of 5 to 7.

### Adverse outcomes

We defined “any adverse outcome” as the presence of any one of the following typical HHT-related severe adverse outcomes: stroke, intracranial hemorrhage (ICH), seizure, anemia (as obtained on medical history), blood transfusion, massive hemoptysis, hemothorax, brain abscess, and death.

### Statistical analysis

We tested for an association between severity score categories and any adverse outcome using Fisher’s exact test. Logistic regression analysis was also performed to test whether moderate and severe severity score categories were significant predictors of an adverse outcome when compared to the mild group. Odds ratios (OR) and 95% confidence intervals (CI) are reported and results are further adjusted for age and gender. An alpha of 0.05 was used to determine statistical significance. All analyses were conducted with Stata version 12.1 [14].

## Results

Of the initial 525 HHT patients, 393 patients (74.9%) were included in the analysis and 132 individuals were excluded (47 pediatric patients, 85 adult patients with incomplete data). The majority of patients were female (65.1%) with a mean age of 49.2 ± 14.3 years. Of those with a confirmed genetic diagnosis of HHT, 54 (33.3%) had an *ALK-1* mutation, 101 (62.4%) had an *ENG* mutation, and 7 (4.3%) had a *SMAD4* mutation. The remaining patients all had a definite clinical diagnosis of HHT, but either had negative HHT gene mutation testing (no mutation identified) or had not undergone genetic testing given their known clinical diagnosis. The majority of patients had a history of recurrent spontaneous epistaxis (381 or 97.0%); GI bleeding was present in 66 (16.8%), PAVMs in 200 (50.9%), BAVMs in 91 (23.2%), and diagnosed liver VMs in 83 (21.1%) patients. The clinical characteristics of HHT patients enrolled in the BVMC study were similar to previously published series [1,11,13,15-23], with the expected exception of a higher prevalence of BAVMs, as per BVMC study recruitment protocol.

**Figure 2:**
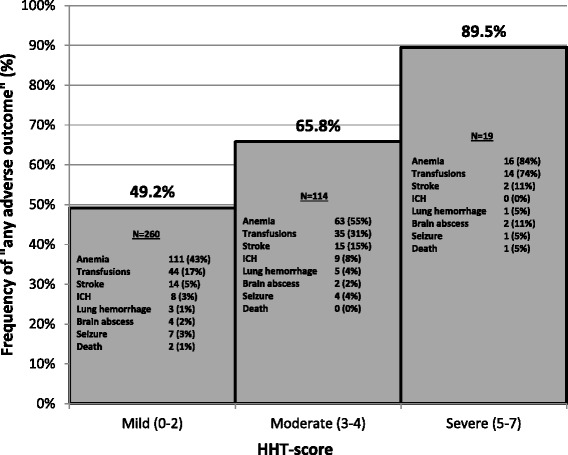
**The frequency of “any adverse outcome” in patients with a mild, moderate and severe HHT-score.** The frequency of “any adverse outcome” is significantly different across the three severity groups (p < 0.001) and there is a clear “dose-dependent” curve. The frequencies of individual adverse outcomes are shown within each bar for the respective severity groups. Absolute frequencies are provided and their relative frequencies, as percentages, in brackets. Note that “lung hemorrhage” includes massive hemoptysis and spontaneous hemothorax.

After HHT-scores were assigned, 260 (66.2%) of the included patients were categorized as having mild disease (HHT-score 0–2), while 114 (29.0%) and 19 (4.8%) patients had moderate (HHT-score 3–4) and severe disease (HHT-score 5–7), respectively. The frequency of any adverse outcome was significantly different across the severity groups (49.6% for mild, 65.8% for moderate, and 89.5% for severe, p < 0.001). The most common adverse outcomes were anemia, need for blood transfusion and stroke for all three severity score categories (Figure 2). Even when removing anemia and blood transfusions as outcomes in the analysis, the frequency of any adverse outcomes remained significantly different across the three severity groups (p < 0.01).

Modeling the severity groups as categorical variables, both the moderate group (OR = 1.95, 95% CI: 1.24-3.08, p = 0.004) and the severe group (OR = 8.63, 95% CI: 1.95-38.11, p = 0.004) were significantly more likely to have had an adverse outcome compared to the mild group. This once again held true even when excluding anemia and blood transfusions from the analysis. In univariable logistic regression analysis, age (per decade) was found to be associated with any adverse outcomes (OR = 1.43, 95% CI: 1.23-1.66, p < 0.001), and gender was not (OR = 1.15, 95% CI: 0.76-1.74, p = 0.517). However, even after adjusting for age and gender, the OR estimates for severity score categories remained significant for the moderate group (OR = 1.84, 95% CI: 1.15-2.95, p = 0.011) and the severe group (OR = 9.16, 95% CI: 1.99-42.09, p = 0.004) compared to the mild severity group, and similar to the unadjusted estimates.

## Discussion

We present here the first overall severity score for HHT, the HHT-score, developed in a well-characterized, multi-center cohort of 393 adult HHT patients. We demonstrate that higher HHT-scores are associated with higher frequencies of adverse outcomes. The HHT-score provides a standardized method of categorizing disease severity in adults and, as such, would help to assess burden of disease and guide clinical trial design.

The frequency of “any adverse outcome” was found to increase significantly with higher HHT-scores, providing preliminary evidence of the validity of the HHT-score for classifying patients by worsening disease severity and for predicting risk of adverse outcome. While increasing age was associated with higher frequency of severe outcomes, neither age nor gender was found to significantly affect the relationship between HHT-score and risk of adverse outcomes in our logistic regression model. Although the frequency of any adverse outcome may appear higher than expected, especially in the mild group, it is easily explained by the study design, given that we collected the adverse outcomes over the patients’ entire lifetime. We justified this approach on the grounds that the disease is present at birth, often many years before diagnosis, and that recorded outcomes should therefore not be restricted to the period of time after diagnosis. In addition, we were reassured by the “dose–response” curve of the frequency of any adverse outcome with HHT-scores, which has good face validity.

One of the strengths of the HHT-score is that it incorporates a comprehensive set of clinical manifestations of the disease from epistaxis to BAVMs, and is simple to apply after routine clinical assessment of an HHT patient. Though it may not seem intuitive to amalgamate everything from epistaxis to BAVMs into a severity score, it is biologically plausible that there are common mechanisms and/or modifiers for the development of hemorrhage from vascular malformations across organs. The observed scores were reasonably distributed across the severity categories, roughly compatible with our clinical experience, though we might have expected a slightly higher percentage (~10%) of patients to have scored in the severe category. We speculate that this may be rectified in the future by including additional criteria for severe organ involvement that were not readily available from the BVMC dataset, such as multiplicity and location of BAVMs, extreme multiplicity of GI telangiectases, presence of juvenile polyposis, pulmonary hypertension, ESS, pulmonary shunt grade, etc. One other strength of the HHT-score is the generalizability of the results; not only were the clinical characteristics of the study cohort similar to those reported in other HHT series in the literature, but the patients also came from multiple centres rather than a single centre.

While data pertaining to pulmonary shunt grades was not collected in this study, its incorporation into future versions of the HHT-score may prove especially useful. There is a growing body of literature on the utility of pulmonary shunt grades in HHT [24-28]. Patients with shunting on bubble echocardiography sometimes do not have detectable PAVMs on CT thorax, and this is thought to be related to either false positive results or microAVMs that are too small to be detected on CT [24,25]. In fact, recent studies have shown that of all HHT patients with grade 1 shunting, which makes up the majority of patients with a positive bubble echo but negative CT, none have PAVMs large enough for embolization [25,26]. Furthermore, individuals with HHT and grade 1 shunting are not at an elevated risk of cerebral complications [27]. This suggests that while some patients in our cohort may have had PAVMs too small to be detected on CT thorax, it should be of little to no clinical consequences. In contrast, patients with grade ≥2 pulmonary shunt grades are significantly more likely to have detectable PAVMs resulting in clinical complications [27], highlighting the possible value of using pulmonary shunt grade as a measure of severe organ involvement in future versions of the HHT-score.

This study is limited by its retrospective nature and the associated biases. Complete data from the medical chart was not always available, resulting in the exclusion of several patients if the maximum possible HHT-score could not be calculated. While the cross-sectional design does not allow us to draw conclusions related to causality, this was not our goal. Given the deliberate design by which patients were recruited (i.e. 3 HHT patients with no BAVMs for every 1 HHT patient with a BAVM), there was a slight overrepresentation of patients with BAVMs. While including more patients with BAVMs may theoretically increase our overall estimates of adverse outcomes, BAVM-related adverse outcomes comprised only a small percentage of the total adverse outcomes for each severity group, and therefore the overrepresentation of BAVMs is unlikely to have influenced our results. Otherwise, the clinical characteristics of our sample were very similar to those of other reported HHT populations. The items, scoring system and score ranges were defined based on expert clinical opinion, rather than a formal item generation process, which might limit generalizability, though the preliminary data is reassuring. Evidently, the HHT-score will require further assessment, including validation in other cohorts and by other investigators and stake-holders, and may require revisions. In addition, there are other clinical factors that may impact disease outcomes in HHT, which could be incorporated into future versions of the HHT-score, such as age, other organs affected by AVMs and further criteria for severe organ involvement as described above. The applicability of the HHT-score to children has also yet been tested and further studies in a pediatric HHT population are needed.

## Conclusions

In summary, we have developed the first overall disease severity score for HHT, and have associated HHT-scores with more frequent adverse outcomes. The HHT-score provides a standardized method of categorizing disease severity in adults and, as such, would help to assess burden of disease and guide clinical trial design. While preliminary results are encouraging, further validation studies are required, as well as prospective studies assessing the predictive power of the score and delineating the HHT-score to children has also not yet been tested and adverse outcomes.

## Abbreviations

ALK-1: Activin A receptor type II-like 1
AVM: Arteriovenous malformation
BVMC: The brain vascular malformation consortium
ENG: Endoglin
ESS: Epistaxis severity score
HHT: Hereditary hemorrhagic telangiectasia
HHT-score: HHT severity score
SMAD4: SMAD family member 4
